# No changes in dietary intake after quitting smoking; a prospective study in Switzerland

**DOI:** 10.1186/s40795-021-00440-4

**Published:** 2021-07-14

**Authors:** Pollyanna Patriota, Idris Guessous, Pedro Marques-Vidal

**Affiliations:** 1 Department of Nutrition, Institute of Health Sciences, Federal University of Triangulo Mineiro, Uberaba, Brazil; 2 Division of primary care medicine, Department of primary care medicine, Geneva university hospitals, Geneva, Switzerland; 3 Department of medicine, internal medicine, Lausanne university hospital (CHUV) and University of Lausanne, rue du Bugnon 46, 1011 Lausanne, Switzerland

**Keywords:** Quitting smoking, Diet, Weight, Prospective study, Epidemiology

## Abstract

**Background:**

After quitting smoking, quitters frequently increase their weight and change their dietary intake. Still, most studies on the topic are over 20 years old and focused on few dietary markers. We analysed the changes in weight and dietary intake after quitting smoking using a large panel of dietary markers.

**Methods:**

Prospective study including 5064 participants, 169 of whom (3.3%) quitted during a median follow-up of 5 years. Dietary intake was assessed using a food frequency questionnaire. Participants were excluded if they lacked dietary data or reported extreme total energy intakes (TEI) < 850 or > 4000 kcal/day.

**Results:**

Data from 128 participants (43.8% women, aged 56.0 ± 10.0 years) were used. After quitting smoking, mean weight increased 2.1 ± 0.7 kg; the majority (58%) of the participants gained over 1 kg, and only 7.1% were on a diet to reduce their weight. Total protein intake increased from (median [interquartile range]) 14.4 [12.9–16.4] to 15.1 [13.4–17.9] % of total energy intake (TEI), *p* = 0.008, while animal protein intake increased from 9.7 [8.0–12.1] to 10.8 [8.5–13.5] %TEI, *p* = 0.011. Fish intake increased from 27 [17–45] to 37 [19–55] g/day, *p* = 0.016 and dairy intake decreased from 177 [94–288] to 150 [77–243] g/day, *p* = 0.009. No other changes were found. Among the 68 (53%) participants who reported time since quitting, quitting for <=1 year led to a decreased consumption of fruits, while the opposite was found for participants who quit for longer than one year. No associations were found between weight or dietary changes and time since quitting.

**Conclusions:**

People who quit smoking tend to gain weight, do not significantly change their dietary intake, and seem to make little effort to prevent weight gain. Systematic dietary support should be provided to all smokers wishing to quit.

**Supplementary Information:**

The online version contains supplementary material available at 10.1186/s40795-021-00440-4.

## Background

Quitting smoking is frequently associated with an increase in weight, which deters smokers from quitting [1]. Although smokers tend to have an unhealthier diet than nonsmokers, there is little information regarding how diet changes after quitting smoking. Almost all studies that assessed dietary changes after quitting smoking reported an increase in total energy intake (TEI) [2, 3] due to an increase in fat [3, 4] and/or carbohydrate [4, 5] intake, namely sugars [3–5], while the consumption of protein remained constant or even decreased [5]. Other studies reported an increase in overall dietary quality after quitting smoking [6], while alcohol consumption either decreased [6] or did not change [4]. Still, most studies have been conducted 30 years ago and, to our knowledge, few of them assessed different types of nutrients such as fatty acids [2] or dietary scores [6].

Hence, we aimed to assess changes in dietary intake after quitting smoking in a population-based sample. We hypothesized that TEI would increase after quitting smoking without significant improvements in dietary quality.

## Materials and methods

### Participants

The Cohorte Lausannoise (CoLaus) study is a population-based study assessing the clinical, biological, and genetic determinants of cardiovascular disease in the city of Lausanne, Switzerland [7]. Briefly, all subjects aged 35 to 75 living in the city of Lausanne were eligible. Participants were included if they consented to participate in the study and were willing to provide a blood sample. Recruitment began in June 2003 and ended in May 2006; the first follow-up was performed between April 2009 and September 2012 and the second follow-up between May 2014 and April 2017. The information collected at follow-ups was similar to the baseline examination, except that dietary assessment was also performed. Hence, for this study, only data from the follow-up examinations (2009–2012 and 2014–2017) were used.

### Quitting smoking

Smoking status (never, former, current) and time since quitting was self-reported. Participants who reported being smokers in the first follow-up and former smokers in the second follow-up were considered as quitters. Participants who reported smoking or having never smoked at both follow-ups were considered as maintainers and never smokers, respectively. Time since quitting was categorized into ≤1 year, > 1 to ≤2 years, and > 2 years.

### Dietary intake

Dietary intake was assessed using a self-administered, semi-quantitative FFQ, which also included portion size. This FFQ has been validated in the Geneva population [8, 9]. Briefly, this FFQ assesses the dietary intake of the previous 4 weeks and consists of 97 different food items that account for more than 90% of the intake of calories, proteins, fat, carbohydrates, alcohol, cholesterol, vitamin D and retinol, and 85% of fiber, carotene, and iron. For each item, consumption frequencies ranging from “less than once during the last 4 weeks” to “2 or more times per day” were provided, and participants indicated the average serving size (smaller, equal or bigger) compared to a reference size. The same food composition database was used throughout the study period.

Participants were dichotomized according to whether they followed the dietary recommendations for fruits, vegetables, meat, fish, and dairy products from the Swiss Society of Nutrition (supplementary Table 1) [10, 11]. As the FFQ queried about fresh and fried fish, two categories were considered: one including and one excluding fried fish.

### Socio-demographic and clinical data

Marital status was categorized as living alone or in a couple. Educational level was categorized into university, high school, apprenticeship, and mandatory. The presence of a diet to reduce weight was assessed by questionnaire.

Body weight and height were measured with participants barefoot and in light indoor clothes. Body mass index (BMI) was computed and categorized into normal (< 25 kg/m^2^), overweight (25–29.9 kg/m^2^) and obese (≥30 kg/m^2^). As only two participants had a BMI < 18.5 kg/m^2^, they were included in the normal weight group. Physical activity was assessed by questionnaire [12] and expressed as energy expenditure (kcal/day) or sedentary status, defined as spending more than 90% of the daily energy in activities below moderate- and high-intensity.

### Inclusion and exclusion criteria

Participants were eligible if they participated in the first and the second follow-ups and reported smoking status.

Participants were excluded if they lacked dietary data or reported extreme total energy intakes (TEI) < 850 or > 4000 kcal/day.

### Statistical analysis

Statistical analyses were performed using Stata version 16.0 for Windows (Stata Corp, College Station, Texas, USA). Descriptive results were expressed as number of participants (percentage) for categorical variables and as average ± standard deviation or median [interquartile range] for continuous variables. Two sets of analyses were conducted. The first one compared the anthropometric and dietary intake of smokers before (period 2009–2012) and after (period 2014–2017) quitting. Bivariate analyses were performed using *McNemar’s* test for categorical variables and paired student’s t-test or sign test for continuous variables. The analyses were further stratified according to gender and BMI category at baseline.

The second set of analyses compared the changes in anthropometric and dietary intake between quitters and maintainers and between quitters and never smokers, matched for gender and age (±1 year). This matching was decided due to differences in dietary intake between genders and age groups [13]. Briefly, for each participant, the differences in anthropometric and dietary intake between the 2014–2017 and the 2009–2012 assessment periods were computed, and between-group comparisons were performed using student’s t-test or Wilcoxon sign test for continuous variables.

Comparison of changes in anthropometric and dietary intake between categories of time since quitting was performed using analysis of variance or Kruskal-Wallis test. Associations between time since quitting and weight gain were assessed using Spearman rank correlation.

Statistical significance was assessed for a two-sided test with *p* < 0.05.

## Results

There were 5064 participants in the first follow-up, of whom 4381 (86.5%) were eligible and 169 were considered as quitters. The selection procedure is summarized in supplementary figure 1. Of the initial 169 participants who quit, 41 (24.3%) were excluded because of missing or extreme dietary intakes, leaving 128 participants (43.8% women, aged 56.0 ± 10.0 years, median follow-up time: 5 years) for analysis. The characteristics of the included and excluded participants are summarized in supplementary Table 2. No differences were found for all variables considered.

### Changes in anthropometry and dietary intake after quitting

**Fig. 1:**
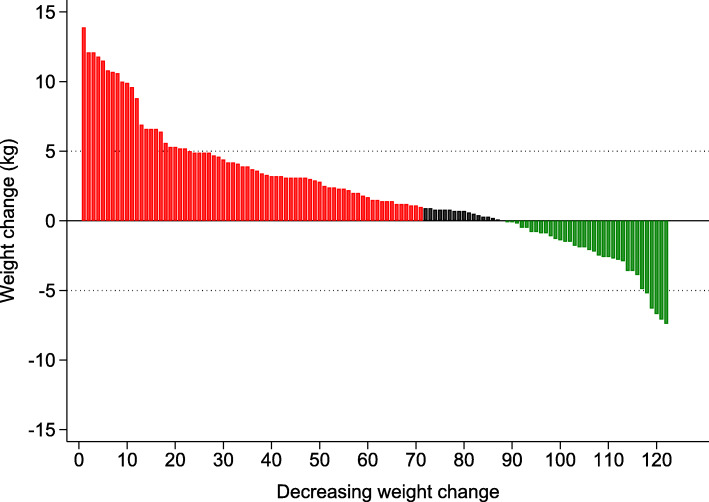
Waterfall plot showing the changes in weight after quitting smoking

The changes in weight and dietary intake before and after quitting are summarized in Table 1. Weight increased 2.1 ± 0.7 kg on average, (+ 0.9 kg/m^2^ increase in BMI) and 70/121 (58%) participants gained at least 1 kg (Fig. 1). Nine (7.1%) participants reported being on a diet to reduce weight after quitting; participants on a diet gained 3.2 ± 6.2 kg, vs. 2.0 ± 4.0 kg for participants who did not diet (*p* = 0.424). Physical activity levels were available for 100 participants; no changes were found in energy expenditure or prevalence of sedentariness after quitting (Table 1). No differences were found regarding total caloric intake before and after quitting (Table 1). Absolute saturated and polyunsaturated fat, calcium, and dairy intake decreased, and fish intake increased significantly after quitting. When expressed as percentage of TEI, saturated fat intake decreased significantly and total and animal protein increased significantly, while no differences were found for all other dietary markers considered (Table 1). With the exception of a higher compliance regarding fish consumption (all types of fish), no significant changes in compliance were found (Table 1).

**Table 1:** Anthropometric, physical activity and dietary data before and after quitting, CoLaus study, Lausanne, Switzerland

	Before quitting	After quitting	*P*-value
Sample size	**128**	**128**	
**Anthropometry** ^c^			
Weight (kg)	72.3 ± 15.8	74.4 ± 16.5	< 0.001 *
Body mass index (kg/m^2^)	25.0 ± 4.4	25.9 ± 4.7	< 0.001 *
**Physical activity** ^d^			
Energy expenditure	2651 [2267; 3064]	2609 [2217; 3198]	0.658
Sedentarity (%)	47 (47.0)	48 (48.0)	1.000
Total energy intake (kcal)	1811 [1396–2234]	1744 [1357–2169]	0.162
**Macronutrients (g/d)**			
Total protein	66 [50; 88]	64 [51; 86]	0.973
Vegetable protein	19 [15; 27]	18 [14; 27]	0.226
Animal protein	45 [33; 60]	46 [33; 60]	0.676
Carbohydrates	200 [152; 268]	188 [147; 253]	0.131
Disaccharides	92 [66; 127]	88 [65; 116]	0.487
Polysaccharides	96 [70; 141]	89 [61; 138]	0.115
Total fat	65 [52; 91]	65 [47; 82]	0.224
SFA	25 [19; 35]	23 [16; 31]	0.008
MUFA	26 [21; 35]	26 [19; 35]	0.698
PUFA	9 [7; 12]	9 [6; 12]	0.036
Alcohol	7 [1; 19]	7 [1; 19]	0.948
Fibre	13 [10–20]	13 [9–19]	0.730
**Macronutrients (% TEI)**			
Total protein	14.4 [12.9–16.4]	15.1 [13.4–17.9]	0.008
Vegetable protein	4.6 [3.8–5.3]	4.4 [3.8–5.3]	0.664
Animal protein	9.7 [8.0–12.1]	10.8 [8.5–13.5]	0.011
Carbohydrates	45.2 [38.8–51.7]	44.1 [39.1–51.0]	0.295
Disaccharides	19.9 [15.3–27.2]	21.0 [15.4–25.6]	0.495
Polysaccharides	22.9 [16.6–27.6]	20.9 [16.6–27.7]	0.199
Total fat	34.5 [29.0–38.8]	34.6 [30.1–39.2]	0.754
SFA	12.9 [10.8–15.3]	12.4 [10.6–14.3]	0.048
MUFA	13.1 [11.0–15.8]	14.3 [11.4–16.4]	0.162
PUFA	4.6 [3.8–5.5]	4.6 [3.8–5.6]	0.935
Alcohol	3.4 [0.6–7.3]	2.8 [0.5–6.5]	0.598
**Micronutrients**			
Cholesterol (mg/d)	289 [218–365]	268 [197–381]	0.978
Calcium (mg/d)	941 [706–1297]	778 [581–1255]	0.028
Iron (mg/d)	10.1 [7.7–12.7]	10.1 [7.5–12.9]	0.931
Vitamin D	1.9 [1.4–2.7]	2.2 [1.4–3.4]	0.066
**Foods (g/day)**			
Dairy	177 [94–288]	150 [77–243]	0.009
Red meat	37 [18–64]	38 [24–64]	0.377
Processed meat	10 [4–19]	10 [3–17]	0.224
Wholegrain	30 [5–76]	27 [6–70]	0.885
Fresh fruits	132 [57–280]	147 [72–264]	0.474
Fresh fruits + fresh juice	169 [72–302]	178 [92–333]	0.097
Any fruit and fruit juice	218 [97–394]	241 [135–370]	0.142
Vegetables	125 [80–197]	134 [89–200]	0.203
Fish, excluding fried	20 [13–35]	28 [15–46]	0.009
Fish, all	27 [17–45]	37 [19–55]	0.016
Ultraprocessed foods	60 [14–148]	54 [7–161]	0.166
**Compliance to guidelines (%)**			
Fruits ≥2/day	41 (32.5)	51 (40.5)	0.143
Vegetables ≥3/day	7 (5.6)	8 (6.4)	1.000
Meat ≤5/week	80 (63.5)	73 (57.9)	0.337
Fish ≥1/week ^a^	82 (65.1)	89 (70.6)	0.296
Fish ≥1/week ^b^	44 (34.9)	58 (46.0)	0.039
Dairy ≥3/day	11 (8.7)	11 (8.7)	1.000

^a^, excluding fried fish; ^b^, all fish. ^c^, for 122 participants; ^d^, for 100 participants

*SFA* saturated fatty acids; *MUFA* monounsaturated fatty acids; *PUFA* polyunsaturated fatty acids; *TEI* total energy intake

Results are expressed as number of participants (column percentage) for categorical variables and as average ± standard deviation or as median [interquartile range] for continuous variables. Between-group comparisons were performed using McNemar’s test for categorical variables and student’s t-test (*) or Wilcoxon’s sign test for continuous variables

When the analyses were stratified according to gender, both genders gained weight after quitting. Regarding dietary intake, similar findings were obtained, but several changes were no longer statistically significant (supplementary Tables 3 and 4).

When the analyses were stratified according to BMI category, weight gain occurred in all categories. Regarding dietary intake, similar findings were obtained, but several changes were no longer statistically significant (supplementary Tables 5 to 7).

### Comparison between smoking groups

The dietary changes between the first and the second follow-ups for “quitters”, “maintainers” and “never smokers” are summarized in Table 2. Quitters had higher weight and BMI gains than maintainers and never smokers. No differences were found regarding changes in energy expenditure (Table 2). Relative to maintainers, quitters increased their total and animal protein intake (expressed as percentage of TEI). Relative to never smokers, quitters decreased their absolute consumption of saturated fat. No differences were found for all other dietary markers considered (Table 2).

**Table 2:** Changes between baseline and follow-up in anthropometry, physical activity and dietary intake between quitters and maintainers, and between quitters and never smokers, matched for gender and age, CoLaus study, Lausanne, Switzerland

	Quitters	Maintainers	Never smokers	***P***-value §	P-value ǂ
Sample size	**128**	**128**	**128**		
**Anthropometry** ^a^					
Weight (kg)	2.2 ± 4.2	0.3 ± 4.4	0.4 ± 4.4	0.004 *	0.003 *
Body mass index (kg/m^2^)	0.92 ± 1.44	0.39 ± 1.50	0.33 ± 1.51	0.013 *	0.002 *
Energy expenditure ^b^	−34 [− 284; 232]	−4 [− 242; 256]	−92 [− 337; 134]	0.441	0.159
Total energy intake (kcal)	−86 [− 450; 316]	10 [− 399; 349]	46 [− 287; 385]	0.739	0.099
**Macronutrients (g/d)**					
Total protein	−1 [− 14; 14]	5 [− 13; 16]	− 4 [− 17; 13]	0.413	0.495
Vegetable protein	− 2 [− 7; 4]	1 [− 3; 5]	− 1 [− 7; 4]	0.116	0.600
Animal protein	0 [− 14; 13]	3 [− 11; 16]	−3 [− 10; 11]	0.556	0.873
Carbohydrates	−13 [− 62; 40]	6 [− 35; 46]	−7 [− 66; 45]	0.226	0.986
Disaccharides	− 4 [− 27; 26]	3 [− 25; 20]	− 1 [− 29; 23]	0.495	0.626
Polysaccharides	−6 [− 35; 24]	5 [− 22; 29]	−1 [− 46; 24]	0.075	0.520
Total fat	−4 [− 20; 14]	4 [− 10; 19]	−2 [− 15; 12]	0.060	0.390
SFA	−2 [−9; 4]	1 [− 5; 7]	− 1 [− 7; 6]	0.013	0.135
MUFA	−1 [− 7; 8]	2 [− 4; 9]	−1 [− 6; 6]	0.123	0.819
PUFA	−1 [−3; 1]	0 [− 2; 3]	−1 [− 2; 2]	0.060	0.446
Alcohol	0 [− 3; 3]	0 [− 3; 5]	0 [− 1; 2]	0.885	0.690
Fibre	0 [− 4; 4]	0 [− 2; 4]	−1 [− 4; 3]	0.335	0.721
**Macronutrients (% TEI)**					
Total protein	0.5 [−1.2; 2.8]	0.1 [− 2; 2]	0.3 [− 1.7; 1.7]	0.188	0.115
Vegetable protein	0 [−0.8; 0.6]	0 [− 0.7; 0.9]	−0.2 [− 0.9; 0.7]	0.641	0.307
Animal protein	0.5 [− 1.2; 3.4]	−0.3 [− 2.3; 2.7]	0 [−1.8; 2.2]	0.199	0.247
Carbohydrates	− 0.6 [− 6.4; 5.0]	−0.6 [− 6.8; 3.4]	−1.2 [− 6.7; 4.7]	0.340	0.432
Disaccharides	0.4 [−3.9; 4.4]	− 1.4 [− 5.7; 3.3]	0.9 [− 4.1; 5.0]	0.027	0.797
Polysaccharides	−0.8 [− 5.3; 3.7]	0.5 [− 5.3; 5.3]	−1.5 [− 6.4; 4.3]	0.462	0.352
Total fat	0.3 [− 4.7; 5.0]	0.3 [− 3.8; 5.3]	1.4 [−3.6; 5.8]	0.399	0.281
SFA	− 0.4 [− 2.9; 1.6]	− 0.1 [− 2.0; 2.4]	0.5 [− 2.0; 2.1]	0.109	0.051
MUFA	0.4 [− 1.4; 2.8]	0.5 [− 2.0; 2.7]	0.6 [− 1.8; 3.0]	0.825	0.638
PUFA	0 [− 0.7; 0.8]	0.1 [− 0.7; 1.0]	0 [− 0.7; 1.0]	0.273	0.556
Alcohol	0 [− 1.6; 1.1]	0 [− 1.7; 2.3]	0 [− 0.5; 0.7]	0.654	0.333
**Micronutrients**					
Cholesterol (mg/d)	−2 [− 78; 84]	40 [− 29; 113]	2 [− 67; 66]	0.021	0.854
Calcium (mg/d)	− 82 [− 399; 165]	53 [− 255; 318]	−37 [− 318; 260]	0.054	0.367
Iron (mg/d)	−0.1 [− 2.1; 2.1]	0.5 [− 1.6; 2.2]	−0.5 [− 3.0; 2.2]	0.638	0.197
Vitamin D	0.1 [−0.5; 1.2]	0.2 [− 0.5; 1.1]	0 [− 0.6; 1.2]	0.982	0.959
**Foods (g/day)**					
Dairy	−27 [−81; 31]	7 [−65; 66]	− 5 [− 66; 56]	0.184	0.215
Red meat	0 [− 14; 18]	4 [− 23; 18]	−5 [− 21; 14]	0.684	0.250
Processed meat	0 [− 7; 4]	0 [− 7; 6]	0 [− 5; 5]	0.459	0.171
Wholegrain	0 [− 18; 22]	0 [− 9; 24]	0 [− 27; 12]	0.284	0.591
Fresh fruits	8 [− 92; 90]	7 [− 47; 81]	− 8 [− 85; 53]	0.721	0.741
Fresh fruits + fresh juice	23 [− 58; 114]	8 [− 49; 102]	− 13 [− 95; 72]	0.585	0.382
Any fruit and fruit juice	22 [− 71; 126]	− 5 [− 87; 80]	2 [− 103; 98]	0.313	0.215
Vegetables	3 [− 35; 59]	2 [−41; 59]	2 [− 43; 61]	0.901	0.714
Fish, excluding fried	2 [−5; 14]	5 [− 5; 17]	0 [− 11; 11]	0.797	0.021
Fish, all	2 [− 10; 17]	4 [− 6; 19]	0 [− 15; 13]	1.000	0.018
Ultraprocessed foods	− 4 [− 46; 29]	0 [− 46; 25]	−2 [− 43; 19]	0.741	0.714

^a^, for 122 participants; ^b^, for 100 participants. §, comparing quitters to maintainers; **ǂ** comparing quitters to never smokers

*SFA* saturated fatty acids; *MUFA* monounsaturated fatty acids; *PUFA* polyunsaturated fatty acids; *TEI* total energy intake

For each participant, the difference between data collected in 2014–2017 and data collected in 2009–2012 were computed Results are expressed as average ± standard deviation or as median [interquartile range]. Between-group comparisons were performed using student’s t-test (*) or Wilcoxon sign test for continuous variables

When the analyses were stratified according to gender, women quitters had higher weight and BMI gains than never smokers, while no differences were found between quitters and maintainers (supplementary Table 8). Male quitters had higher weight and BMI gains than maintainers and never smokers (supplementary Table 9). Regarding dietary intake, women quitters increased their total and animal protein intake (expressed as percentage of TEI) relative to never quitters, while no differences were found in men (supplementary Tables 8 and 9).

### Association with time since quitting

Among the 128 participants, time since quitting was reported by 68 (53%): median and [interquartile range] 2.1 [1.0–3.3 years]. The dietary changes according to time since quitting are provided in supplementary Table 10. Participants who had quit for ≤1 year decreased their consumption of fruits, while the opposite was found for participants who had quit for longer than one year. No associations were found between weight or dietary changes and time since quitting (supplementary Table 11).

## Discussion

To our knowledge, this is the first study assessing weight gain and dietary changes among smoking quitters conducted in Switzerland. Our results show that quitting smoking was associated with a mean weight change of 2.1 kg, corresponding to an increase of 0.9 BMI units over a median follow-up of 5 years. Conversely, and contrary to our initial hypothesis, no changes in reported total caloric intake were found.

### Changes in dietary intake after quitting

Weight gain increased by an average of 2.1 kg, a finding in line with a meta-analysis and systematic review involving diverse populations around the world [1]; noteworthy, the authors failed to find studies conducted in Switzerland. Indeed, weight gain is a common occurrence after quitting smoking and is one of the reasons why many quitters tend to relapse [1]. Still, adequate dietary management after quitting has been shown to prevent weight gain [14]. In this study, only nine participants who quit reported being on a diet, and achieved no weight loss compared to the non-dieting participants. Hence, our results suggest that effective dietary support to control weight is not provided to quitters on a regular basis.

No changes in total energy expenditure or prevalence of sedentariness were found. Our results replicate those of a previous study, where no changes in physical activity were noted after quitting [15]. Still, physical activity data was queried via questionnaire and reporting biases cannot be excluded; further studies assessing physical activity via more precise methods (i.e. accelerometry) would be welcomed.

Contrary to what was hypothesized, no increase in reported total energy intake was found among quitters. Our findings do not replicate those from studies conducted 30 years ago [2, 3] but are in line with a recent Australian study, where weight gain associated with smoking cessation was not explained by worsening dietary and physical activity behaviors [6]. A possible explanation for the results of the Australian study is that the authors assessed dietary intake one year after quitting, and is has been shown that quitters increase their energy intake shortly after quitting [2, 4]. Still, in our study, no differences in energy intake were found between different quitting periods. Hence, our results suggest that quitters do not increase their energy intake in the first year after quitting. Still, our sample size was small and it would be of interest to replicate the study in a larger sample.

Dietary intake of quitters changed little before and after quitting, and findings were replicated after stratifying on gender or on BMI category. The absolute decrease in total and saturated fat intake (as kcal) was small and clinically irrelevant, as it corresponded to 2 g of fat per day. Hence, our results suggest that the changes in dietary intake observed among quitters do not contribute to the weight gain.

### Comparison between smoking groups

Quitters had a higher weight gain than maintainers and never smokers, suggesting that the increase was not due to aging. No significant differences were found regarding changes in the diet. Women quitters showed a higher intake of total and animal protein compared to never smokers, while no differences were found between quitters and maintainers. Our results do not replicate those of a previous study where women who quit had higher energy and lower fat intake than did women who continued smoking [16]. Still, several studies indicate that the benefits of quitting smoking outweigh those of weight gain [17]. Hence, quitters should refrain from smoking, even at the expense of an increase in body weight. Optimally, people desiring to quit should be given lifestyle advice to prevent and control weight gain after smoking cessation.

### Association with time since quitting

Few changes in anthropometric or dietary intake were found according to time since quitting. The exception was fruits, the consumption of which decreased participants who had quit for less than one year but increased afterwards. A previous study showed an inverse association between fruit and vegetable consumption and weight gain among people who quit smoking [18], while no such association was found in another [6]. Although we cannot rule out that this association might have occurred by chance, negative albeit statistically nonsignificant correlations were found between changes in fruit or vegetable intake and weight (Supplementary Table 11). Still, our results suggest that dietary intake does not change after quitting smoking and is not associated with weight gain, a finding also reported elsewhere [6].

### Possible mediators

In this study, neither physical activity nor dietary intake changes could explain the weight gain that occurred in the majority of participants. It has been suggested that dietary changes occur during the first 6 months and return to baseline levels by one year [4]. This could explain the lack of differences regarding dietary intake, as most quitters had quit for over one year. Another possible explanation would be changes in intestinal microbiota after quitting [19] but further studies are needed to better identify the determinants of weight gain after quitting.

Several studies have suggested that low socio-economic status (SES) is associated with a low likelihood of quitting smoking [20], although the opposite trend (i.e. lower income people having a higher likelihood of making a quit attempt) was reported in a German study [21]. In a previous paper, we found no clear association between educational level and quitting, although a trend (*p* = 0.064) towards lower quitting rates with lower educational levels was found [22]. Hence, it is possible that the participants who quit had a higher SES and thus a healthier lifestyle, which did not change significantly after quitting.

### Study limitations

This study has several limitations. Firstly, the sample size was small, leading to a low statistical power. Hence, it is likely that some changes in dietary intake have gone unnoticed; still, the sample size is comparable to another study (*N* = 124) [6]. Secondly, timing of smoking cessation was unavailable for almost half of the participants. Hence, it was not possible to assess if the changes in dietary intake occurred during the first years of smoking cessation. Thirdly, a sizable fraction of smokers was excluded, which might limit the generalizability of the findings. Still, this was necessary as many excluded participants had either no or misreported dietary data. Fourthly, the same food composition database was used at both time points, and possible changes in the composition of some foods could have occurred. Further studies should try to assess this point. Finally, our study was conducted in geographically limited population and results might not be applicable in other settings.

We conclude that quitting smoking is associated with weight gain in most quitters and is not accompanied by significant changes in dietary intake. Systematic dietary support should be provided to all smokers wishing to quit.

## Supplementary Information

**Additional file 1 Supplementary Table 1**: food consumption guidelines of the Swiss society of nutrition. **Supplementary Table 2**. Characteristics of included and excluded participants, CoLaus study, Lausanne, Switzerland. **Supplementary Table 3.** Anthropometric, physical activity and dietary data before and after quitting, CoLaus study, Lausanne, Switzerland, women. **Supplementary Table 4.** Anthropometric, physical activity and dietary data before and after quitting, CoLaus study, Lausanne, Switzerland, men. **Supplementary Table 5.** Anthropometric, physical activity and dietary data before and after quitting, CoLaus study, Lausanne, Switzerland, normal weight participants. **Supplementary Table 6.** Anthropometric, physical activity and dietary data before and after quitting, CoLaus study, Lausanne, Switzerland, overweight participants. **Supplementary Table 7.** Anthropometric, physical activity and dietary data before and after quitting, CoLaus study, Lausanne, Switzerland, obese participants. **Supplementary Table 8.** Changes between baseline and follow-up in anthropometry, physical activity and dietary intake between quitters and maintainers, and between quitters and never smokers, matched for gender and age, CoLaus study, Lausanne, Switzerland, women. **Supplementary Table 9.** Changes between baseline and follow-up in anthropometry, physical activity and dietary intake between quitters and maintainers, and between quitters and never smokers, matched for gender and age, CoLaus study, Lausanne, Switzerland, men. **Supplementary Table 10.** Changes between baseline and follow-up in anthropometry, physical activity and dietary intake according to time since quitting, CoLaus study, Lausanne, Switzerland. **Supplementary Table 11.** Correlations between changes between baseline and follow-up in anthropometry and dietary and time since quitting or weight changes, CoLaus study, Lausanne, Switzerland.

**Additional file 2.** Supplementary Figure 1.

## Data Availability

The datasets analysed for this study are not readily available because neither the participants nor the Ethical authorities consented for their publication. Requests to access the datasets should be directed via the CoLaus| website www.colaus-psycolaus.ch/professionals/how-to-collaborate/.

## Abbreviations

TEI: Total Energy Intake
CoLaus: Cohorte Lausannoise
FFQ: Food Frequency Questionnaire
BMI: Body mass index
