# Supplementary material for: No changes in dietary intake after quitting smoking; a prospective study in Switzerland
Source: BMC Nutr. 2021 Jul 14;7:34. doi: 10.1186/s40795-021-00440-4 (PMC8278689; doi:10.1186/s40795-021-00440-4)
Supplement: Supplementary file 1 — Additional file 1 Supplementary Table 1: food consumption guidelines of the Swiss society of nutrition. Supplementary Table 2. Characteristics of included and excluded participants, CoLaus study, Lausanne, Switzerland. Supplementary Table 3. Anthropometric, physical activity and dietary data before and after quitting, CoLaus study, Lausanne, Switzerland, women. Supplementary Table 4. Anthropometric, physical activity and dietary data before and after quitting, CoLaus study, Lausanne, Switzerland, men. Supplementary Table 5. Anthropometric, physical activity and dietary data before and after quitting, CoLaus study, Lausanne, Switzerland, normal weight participants. Supplementary Table 6. Anthropometric, physical activity and dietary data before and after quitting, CoLaus study, Lausanne, Switzerland, overweight participants. Supplementary Table 7. Anthropometric, physical activity and dietary data before and after quitting, CoLaus study, Lausanne, Switzerland, obese participants. Supplementary Table 8. Changes between baseline and follow-up in anthropometry, physical activity and dietary intake between quitters and maintainers, and between quitters and never smokers, matched for gender and age, CoLaus study, Lausanne, Switzerland, women. Supplementary Table 9. Changes between baseline and follow-up in anthropometry, physical activity and dietary intake between quitters and maintainers, and between quitters and never smokers, matched for gender and age, CoLaus study, Lausanne, Switzerland, men. Supplementary Table 10. Changes between baseline and follow-up in anthropometry, physical activity and dietary intake according to time since quitting, CoLaus study, Lausanne, Switzerland. Supplementary Table 11. Correlations between changes between baseline and follow-up in anthropometry and dietary and time since quitting or weight changes, CoLaus study, Lausanne, Switzerland. [file 40795_2021_440_MOESM1_ESM.docx]

Supplementary Material

No changes in dietary intake after quitting smoking; a prospective study in Switzerland

**Pollyanna Patriota^1^, Idris Guessous^2^, ^*^Pedro Marques-Vidal^3^**

^1^ Department of Nutrition, Institute of Health Sciences, Federal University of Triangulo Mineiro, Brazil

^2^ Division of primary care medicine, Department of primary care medicine, Geneva university hospitals, Geneva, Switzerland

^3^ Department of medicine, internal medicine, Lausanne university hospital (CHUV), rue du Bugnon,46, 1011 Lausanne, Switzerland

*** Correspondence:**Pedro Marques-Vidal
[Pedro-Manuel.Marques-Vidal@chuv.ch](mailto:Pedro-Manuel.Marques-Vidal@chuv.ch)

# Supplementary figure 1 legend

Selection procedure.

Other smoking categories include former smokers at both follow-ups and former smokers or never smokers who started smoking between first and second follow-ups.

# Supplementary Data

**Supplementary table 1**: food consumption guidelines of the Swiss society of nutrition

| **Food item** | **Recommendation** |
| --- | --- |
| Fruits | ≥2 portions/day |
| Vegetables | ≥3 portions/day |
| Meat (all types) | ≤5 portions/week |
| Fish | ≥1 portions/week |
| Dairy products | ≥3 portions/day |

**Supplementary table 2**. Characteristics of included and excluded participants, CoLaus study, Lausanne, Switzerland.

|  | **Included** | **Excluded** | **P-value** |
| --- | --- | --- | --- |
| Sample size | 128 | 41 |  |
| Women (%) | 56 (43.8) | 24 (58.5) | 0.099 |
| Age (years) | 56.0 ± 10.0 | 55.5 ± 10.8 | 0.778 |
| Age groups (%) |  |  | 0.755 |
| [40-50[ | 43 (33.6) | 15 (36.6) |  |
| [50-60[ | 46 (35.9) | 14 (34.2) |  |
| [60-70[ | 26 (20.3) | 6 (14.6) |  |
| [70+] | 13 (10.2) | 6 (14.6) |  |
| Married (%) | 68 (53.1) | 22 (53.7) | 0.952 |
| **Before quitting** |  |  |  |
| Weight (kg) | 72.3 ± 15.8 | 69.4 ± 14.5 | 0.309 |
| Body mass index (kg/m^2^) § | 25.0 ± 4.4 | 25.4 ± 3.9 | 0.550 |
| Body mass index groups (%) |  |  | 0.451 |
| Normal | 69 (55.2) | 18 (43.9) |  |
| Overweight | 42 (33.6) | 17 (41.5) |  |
| Obese | 14 (11.2) | 6 (14.6) |  |
| **After quitting** |  |  |  |
| Weight (kg) | 74.3 ± 16.6 | 72.7 ± 16.0 | 0.581 |
| Body mass index (kg/m^2^) § | 25.9 ± 4.7 | 26.8 ± 4.9 | 0.308 |

§ N=122 included participants. Results are expressed as number of participants (column percentage) for categorical variables and as average±standard deviation for continuous variables. Between-group comparisons were performed using chi-square test for categorical variables and student’s t-test for continuous variables

**Supplementary table 3.** Anthropometric, physical activity and dietary data before and after quitting, CoLaus study, Lausanne, Switzerland, women.

|  | **Before** | **After** | **P-value** |
| --- | --- | --- | --- |
| Sample size | **56** | **56** |  |
| **Anthropometry** ‡ |  |  |  |
| Weight (kg) | 61.6 ± 10.3 | 63.0 ± 11.2 | 0.010 * |
| Body mass index (kg/m^2^) | 23.4 ± 3.7 | 24.1 ± 4.1 | <0.001 * |
| **Physical activity †** |  |  |  |
| Energy expenditure | 2266 [2030 ; 2522] | 2249 [2037 ; 2524] | 0.797 |
| Sedentarity (%) | 23 (53.5) | 23 (53.5) | 1.000 |
| Total energy intake (kcal) | 1534 [1218 ; 1959] | 1520 [1141 ; 1970] | 0.619 |
| **Macronutrients (g/d)** |  |  |  |
| Total protein | 58 [46 ; 74] | 57 [47 ; 78] | 0.312 |
| Vegetable protein | 18 [14 ; 24] | 18 [13 ; 25] | 0.659 |
| Animal protein | 39 [28 ; 51] | 38 [29 ; 56] | 0.176 |
| Carbohydrates | 180 [140 ; 251] | 175 [123 ; 236] | 0.201 |
| Disaccharides | 91 [66 ; 118] | 86 [59 ; 113] | 0.261 |
| Polysaccharides | 81 [57 ; 107] | 76 [54 ; 115] | 0.429 |
| Total fat | 59 [46 ; 79] | 55 [43 ; 77] | 0.887 |
| SFA | 22 [17 ; 30] | 19 [14 ; 29] | 0.401 |
| MUFA | 24 [19 ; 31] | 25 [18 ; 33] | 0.768 |
| PUFA | 8 [6 ; 10] | 7 [6 ; 10] | 0.454 |
| Alcohol | 3 [0 ; 7] | 3 [0 ; 7] | 0.642 |
| Fibre | 15 [10 ; 20] | 14 [10 ; 20] | 0.781 |
| **Macronutrients (% TEI)** |  |  |  |
| Total protein | 14.5 [13.3 ; 16.5] | 15.3 [13.8 ; 18.5] | 0.007 |
| Vegetable protein | 4.6 [4.0 ; 5.5] | 4.7 [4.1 ; 5.6] | 0.755 |
| Animal protein | 9.6 [8.0 ; 11.8] | 10.7 [8.6 ; 13.5] | 0.025 |
| Carbohydrates | 47.0 [40.9 ; 54.0] | 44.1 [39.9 ; 51.7] | 0.198 |
| Disaccharides | 22.2 [16.4 ; 29.9] | 23.4 [17.3 ; 26.1] | 0.379 |
| Polysaccharides | 21.6 [15.7 ; 24.7] | 20.6 [16.9 ; 24.6] | 0.677 |
| Total fat | 35.0 [28.5 ; 40.0] | 35.1 [31.2 ; 40.4] | 0.755 |
| SFA | 12.6 [9.6 ; 15.0] | 11.8 [9.7 ; 14.3] | 0.636 |
| MUFA | 13.5 [11.3 ; 16.4] | 14.7 [11.7 ; 17.1] | 0.401 |
| PUFA | 4.8 [3.6 ; 5.9] | 4.9 [3.7 ; 5.6] | 0.958 |
| Alcohol |  |  | 0.572 |
| **Micronutrients** |  |  |  |
| Cholesterol (mg/d) | 247 [165 ; 305] | 241 [186 ; 347] | 0.108 |
| Calcium (mg/d) | 836 [625 ; 1183] | 696 [520 ; 1249] | 0.370 |
| Iron (mg/d) | 9.7 [6.8 ; 11.3] | 9.2 [7.3 ; 11.7] | 0.585 |
| Vitamin D | 2 [1.3 ; 2.8] | 2.4 [1.4 ; 4.4] | 0.037 |
| **Foods (g/day)** |  |  |  |
| Dairy | 157 [97 ; 298] | 156 [56 ; 261] | 0.032 |
| Red meat | 24 [13 ; 41] | 30 [17 ; 44] | 0.526 |
| Processed meat | 5 [3 ; 12] | 5 [0 ; 12] | 0.841 |
| Wholegrain | 40 [11 ; 77] | 51 [10 ; 76] | 0.870 |
| Fresh fruits | 204 [76 ; 345] | 200 [83 ; 281] | 0.701 |
| Fresh fruits + fresh juice | 243 [103 ; 386] | 261 [135 ; 371] | 0.900 |
| Any fruit and fruit juice | 322 [134 ; 524] | 324 [167 ; 414] | 0.505 |
| Vegetables | 158 [94 ; 222] | 167 [114 ; 263] | 0.031 |
| Fish, excluding fried | 24 [11 ; 38] | 32 [13 ; 47] | 0.035 |
| Fish, all | 29 [16 ; 46] | 39 [16 ; 58] | 0.053 |
| Ultraprocessed foods | 38 [8 ; 107] | 28 [5 ; 125] | 0.137 |
| **Compliance to guidelines (%)** |  |  |  |
| Fruits ≥2/day | 27 (48.2) | 32 (57.1) | 0.359 |
| Vegetables ≥3/day | 6 (10.7) | 6 (10.7) | 1.000 |
| Meat ≤5/week | 43 (76.8) | 41 (73.2) | 0.754 |
| Fish ≥1/week ^a^ | 38 (67.9) | 38 (67.9) | 1.000 |
| Fish ≥1/week ^b^ | 26 (46.4) | 29 (51.8) | 0.467 |
| Dairy ≥3/day | 8 (14.3) | 7 (12.5) | 1.000 |

^a^, excluding fried fish; ^b^, all fish; ‡ on 53 participants; † on 43 participants

SFA, saturated fatty acids; MUFA, monounsaturated fatty acids; PUFA, polyunsaturated fatty acids; TEI, total energy intake.

Results are expressed as number of participants (column percentage) for categorical variables and as average±standard deviation or as median [interquartile range] for continuous variables. Between-group comparisons were performed using McNemar’s test for categorical variables and student’s t-test (*) or Wilcoxon’s sign test for continuous variables.

**Supplementary table 4.** Anthropometric, physical activity and dietary data before and after quitting, CoLaus study, Lausanne, Switzerland, men.

|  | **Before** | **After** | **P-value** |
| --- | --- | --- | --- |
| Sample size | **72** | **72** |  |
| **Anthropometry** ‡ |  |  |  |
| Weight (kg) | 80.5 ± 14.4 | 83.2 ± 14.5 | <0.001 * |
| Body mass index (kg/m^2^) | 26.2 ± 4.6 | 27.2 ± 4.7 | <0.001 * |
| **Physical activity †** |  |  |  |
| Energy expenditure | 3010 [2652 ; 3552] | 3054 [2603 ; 3640] | 0.523 |
| Sedentarity (%) | 24 (42.1) | 25 (43.9) | 1.000 |
| Total energy intake (kcal) | 2000 [1586 - 2414] | 1909 [1533 - 2375] | 0.186 |
| **Macronutrients (g/d)** |  |  |  |
| Total protein | 72 [57 ; 89] | 71 [57 ; 88] | 0.361 |
| Vegetable protein | 22 [16 ; 29] | 18 [14 ; 27] | 0.220 |
| Animal protein | 50 [37 ; 66] | 50 [38 ; 62] | 0.594 |
| Carbohydrates | 220 [161 ; 286] | 199 [157 ; 265] | 0.327 |
| Disaccharides | 92 [66 ; 133] | 90 [69 ; 119] | 0.953 |
| Polysaccharides | 119 [82 ; 157] | 94 [70 ; 148] | 0.156 |
| Total fat | 76 [58 ; 96] | 72 [51 ; 87] | 0.156 |
| SFA | 31 [22 ; 40] | 26 [19 ; 33] | 0.005 |
| MUFA | 28 [22 ; 37] | 27 [19 ; 35] | 0.417 |
| PUFA | 10 [7 ; 14] | 10 [7 ; 13] | 0.044 |
| Alcohol | 15 [5 ; 28] | 12 [3 ; 27] | 0.742 |
| Fibre | 13 [9 - 20] | 12 [9 - 19] | 0.761 |
| **Macronutrients (% TEI)** |  |  |  |
| Total protein | 14.3 [12.9 - 16.3] | 14.9 [13.1 - 17.6] | 0.228 |
| Vegetable protein | 4.5 [3.7 - 5.0] | 4.3 [3.5 - 5.1] | 0.404 |
| Animal protein | 9.7 [8.0 - 12.3] | 11.0 [8.5 - 13.5] | 0.127 |
| Carbohydrates | 44.0 [38.0 - 49.1] | 43.6 [37.1 - 49.8] | 0.900 |
| Disaccharides | 18.9 [15 - 23.1] | 18.8 [15.1 - 24.3] | 0.087 |
| Polysaccharides | 25.1 [18.1 - 28.6] | 21.7 [16.4 - 29.4] | 0.176 |
| Total fat | 34.1 [29.6 - 37.9] | 33.9 [29.3 - 38.0] | 0.953 |
| SFA | 13.2 [11.2 - 15.4] | 12.8 [10.8 - 14.3] | 0.026 |
| MUFA | 12.7 [10.9 - 15.3] | 13.7 [11.1 - 16.2] | 0.244 |
| PUFA | 4.5 [3.8 - 5.3] | 4.5 [3.8 - 5.5] | 0.900 |
| Alcohol | 4.6 [2.2 - 9.4] | 5.1 [1.0 - 8.9] | 0.309 |
| **Micronutrients** |  |  |  |
| Cholesterol (mg/d) | 322 [264 - 404] | 296 [233 - 395] | 0.184 |
| Calcium (mg/d) | 1069 [725 - 1380] | 836 [610 - 1273] | 0.039 |
| Iron (mg/d) | 10.9 [8.6 - 13.7] | 11.1 [8.5 - 13.3] | 0.740 |
| Vitamin D | 1.9 [1.5 - 2.7] | 2.1 [1.3 - 3.4] | 0.586 |
| **Foods (g/day)** |  |  |  |
| Dairy | 187 [88 - 285] | 149 [93 - 232] | 0.129 |
| Red meat | 46 [28 - 72] | 50 [27 - 74] | 0.534 |
| Processed meat | 15 [7 - 23] | 12 [7 - 19] | 0.215 |
| Wholegrain | 23 [2 - 62] | 16 [4 - 50] | 0.972 |
| Fresh fruits | 96 [45 - 192] | 129 [57 - 223] | 0.140 |
| Fresh fruits + fresh juice | 110 [58 - 213] | 138 [65 - 269] | 0.021 |
| Any fruit and fruit juice | 181 [76 - 309] | 201 [124 - 337] | 0.005 |
| Vegetables | 114 [74 - 165] | 111 [73 - 156] | 0.699 |
| Fish, excluding fried | 18 [13 - 32] | 27 [15 - 40] | 0.109 |
| Fish, all | 26 [18 - 41] | 35 [19 - 52] | 0.137 |
| Ultraprocessed foods | 78 [24 - 167] | 72 [26 - 188] | 0.452 |
| **Compliance to guidelines (%)** ‖ |  |  |  |
| Fruits ≥2/day | 14 (20) | 19 (27.1) | 0.359 |
| Vegetables ≥3/day | 1 (1.4) | 2 (2.9) | 1.000 |
| Meat ≤5/week | 37 (52.9) | 32 (45.7) | 0.458 |
| Fish ≥1/week ^a^ | 44 (62.9) | 51 (72.9) | 0.210 |
| Fish ≥1/week ^b^ | 18 (25.7) | 29 (41.4) | 0.035 |
| Dairy ≥3/day | 3 (4.3) | 4 (5.7) | 1.000 |

^a^, excluding fried fish; ^b^, all fish; ‡ on 69 participants; †, on 57 participants; ‖, on 70 participants.

SFA, saturated fatty acids; MUFA, monounsaturated fatty acids; PUFA, polyunsaturated fatty acids; TEI, total energy intake.

Results are expressed as number of participants (column percentage) for categorical variables and as average±standard deviation or as median [interquartile range] for continuous variables. Between-group comparisons were performed using McNemar’s test for categorical variables and student’s t-test (*) or Wilcoxon’s sign test for continuous variables.

**Supplementary table 5.** Anthropometric, physical activity and dietary data before and after quitting, CoLaus study, Lausanne, Switzerland, normal weight participants.

|  | **Before** | **After** | **P-value** |
| --- | --- | --- | --- |
| Sample size | **69** | **69** |  |
| **Anthropometry** ‡ |  |  |  |
| Weight (kg) | 62.2 ± 9.1 | 64.2 ± 9.9 | <0.001 * |
| Body mass index (kg/m^2^) | 22.0 ± 1.9 | 22.9 ± 2.2 | <0.001 |
| **Physical activity †** |  |  |  |
| Energy expenditure | 2449 [2061 ; 2833] | 2421 [2142 ; 2943] | 0.370 |
| Sedentarity (%) | 24 (45.3) | 25 (47.2) | 1.000 |
| Total energy intake (kcal) | 1648 [1361 ; 2207] | 1626 [1347 ; 2261] | 0.717 |
| **Macronutrients (g/d)** |  |  |  |
| Total protein | 62 [50 ; 88] | 64 [49 ; 90] | 0.238 |
| Vegetable protein | 19 [14 ; 27] | 19 [15 ; 27] | 0.817 |
| Animal protein | 43 [33 ; 60] | 46 [31 ; 61] | 0.165 |
| Carbohydrates | 199 [153 ; 271] | 186 [148 ; 250] | 0.329 |
| Disaccharides | 91 [68 ; 118] | 89 [61 ; 114] | 0.476 |
| Polysaccharides | 94 [66 ; 138] | 91 [67 ; 140] | 0.560 |
| Total fat | 62 [50 ; 85] | 67 [49 ; 89] | 0.708 |
| SFA | 23 [18 ; 33] | 23 [17 ; 30] | 0.353 |
| MUFA | 25 [20 ; 34] | 27 [19 ; 36] | 0.461 |
| PUFA | 9 [7 ; 11] | 9 [7 ; 12] | 0.835 |
| Alcohol | 5 [1 ; 16] | 5 [1 ; 13] | 0.715 |
| Fibre | 14 [10 ; 20] | 14 [10 ; 19] | 0.896 |
| **Macronutrients (% cal)** |  |  |  |
| Total protein | 14.4 [13.3 ; 16.8] | 15.3 [13.5 ; 18.6] | 0.022 |
| Vegetable protein | 4.7 [4.0 ; 5.3] | 4.6 [4 ; 5.4] | 0.576 |
| Animal protein | 9.6 [8.4 ; 12.3] | 10.8 [8.5 ; 14.2] | 0.035 |
| Carbohydrates | 45.5 [40.6 ; 51.7] | 44.1 [40.1 ; 50.4] | 0.115 |
| Disaccharides | 19.5 [15.4 ; 28.5] | 20.8 [15.5 ; 24.8] | 0.704 |
| Polysaccharides | 23.2 [18.8 ; 27.0] | 22.5 [17.5 ; 27.6] | 0.905 |
| Total fat | 35.2 [28.7 ; 38.4] | 35.0 [30.6 ; 40.0] | 0.312 |
| SFA | 13 [10.8 ; 15.4] | 12.5 [11 ; 14.3] | 0.436 |
| MUFA | 13.3 [11.1 ; 15.6] | 14.5 [11.6 ; 16.5] | 0.102 |
| PUFA | 4.3 [3.6 ; 5.4] | 4.6 [3.7 ; 5.5] | 0.258 |
| Alcohol | 2.0 [0.5 ; 6.0] | 2.2 [0.4 ; 5.6] | 0.699 |
| **Micronutrients** |  |  |  |
| Cholesterol (mg/d) | 288 [219 ; 357] | 285 [208 ; 409] | 0.233 |
| Calcium (mg/d) | 916 [705 ; 1251] | 760 [584 ; 1259] | 0.200 |
| Iron (mg/d) | 10.0 [7.4 ; 11.6] | 10.3 [7.4 ; 12.9] | 0.356 |
| Vitamin D | 2.1 [1.3 ; 2.7] | 2.2 [1.4 ; 3.6] | 0.070 |
| **Foods (g/day)** |  |  |  |
| Dairy | 146 [83 ; 282] | 146 [69 ; 258] | 0.048 |
| Red meat | 36 [15 ; 62] | 34 [19 ; 55] | 0.857 |
| Processed meat | 7 [3 ; 14] | 7 [2 ; 12] | 0.654 |
| Wholegrain | 37 [13 ; 90] | 45 [13 ; 81] | 0.809 |
| Fresh fruits | 130 [56 ; 280] | 147 [78 ; 246] | 0.566 |
| Fresh fruits + fresh juice | 159 [74 ; 301] | 170 [94 ; 324] | 0.263 |
| Any fruit and fruit juice | 215 [95 ; 387] | 232 [135 ; 368] | 0.509 |
| Vegetables | 134 [84 ; 211] | 147 [89 ; 258] | 0.040 |
| Fish, excluding fried | 24 [13 ; 38] | 31 [13 ; 46] | 0.027 |
| Fish, all | 30 [16 ; 47] | 38 [16 ; 53] | 0.058 |
| Ultraprocessed foods | 45 [11 ; 120] | 38 [7 ; 144] | 0.147 |
| **Compliance to guidelines (%)** |  |  |  |
| Fruits ≥2/day | 22 (31.9) | 28 (40.6) | 0.238 |
| Vegetables ≥3/day | 5 (7.3) | 8 (11.6) | 0.508 |
| Meat ≤5/week | 48 (69.6) | 42 (60.9) | 0.210 |
| Fish ≥1/week ^a^ | 45 (65.2) | 47 (68.1) | 0.791 |
| Fish ≥1/week ^b^ | 28 (40.6) | 34 (49.3) | 0.238 |
| Dairy ≥3/day | 9 (13.0) | 8 (11.6) | 1.000 |

^a^, excluding fried fish; ^b^, all fish; ‡ on 68 participants; **†** on 53 participants.

SFA, saturated fatty acids; MUFA, monounsaturated fatty acids; PUFA, polyunsaturated fatty acids; TEI, total energy intake.

Results are expressed as number of participants (column percentage) for categorical variables and as average±standard deviation or as median [interquartile range] for continuous variables. Between-group comparisons were performed using McNemar’s test for categorical variables and student’s t-test (*) or Wilcoxon’s sign test for continuous variables.

**Supplementary table 6.** Anthropometric, physical activity and dietary data before and after quitting, CoLaus study, Lausanne, Switzerland, overweight participants.

|  | **Before** | **After** | **P-value** |
| --- | --- | --- | --- |
| Sample size | 42 | 42 |  |
| **Anthropometry** ‡ |  |  |  |
| Weight (kg) | 81.4 ± 7.8 | 83.3 ± 9.6 | 0.019 * |
| Body mass index (kg/m^2^) | 26.9 ± 1.4 | 27.7 ± 1.7 | 0.003 * |
| **Physical activity †** |  |  |  |
| Energy expenditure | 2897 [2556 ; 3463] | 2836 [2472 ; 3640] | 0.326 |
| Sedentarity (%) | 15 (44.1) | 14 (41.2) | 1.000 |
| Total energy intake (kcal) | 1962 [1639 ; 2345] | 1774 [1438 ; 2149] | 0.119 |
| **Macronutrients (g/d)** |  |  |  |
| Total protein | 72 [58 ; 88] | 65 [54 ; 84] | 0.116 |
| Vegetable protein | 22 [15 ; 28] | 17 [13 ; 26] | 0.122 |
| Animal protein | 49 [35 ; 65] | 46 [35 ; 58] | 0.263 |
| Carbohydrates | 217 [163 ; 259] | 195 [156 ; 261] | 0.243 |
| Disaccharides | 97 [70 ; 126] | 90 [77 ; 116] | 0.814 |
| Polysaccharides | 104 [75 ; 150] | 84 [62 ; 137] | 0.060 |
| Total fat | 73 [61 ; 94] | 70 [49 ; 81] | 0.029 |
| SFA | 30 [23 ; 36] | 25 [16 ; 32] | 0.012 |
| MUFA | 28 [23 ; 36] | 26 [20 ; 35] | 0.141 |
| PUFA | 11 [8 ; 15] | 9 [6 ; 12] | 0.002 |
| Alcohol | 11 [3 ; 22] | 8 [2 ; 19] | 0.643 |
| Fibre | 14 [10 ; 20] | 12 [9 ; 19] | 0.243 |
| **Macronutrients (% TEI)** |  |  |  |
| Total protein | 14.2 [12.7 ; 15.8] | 15 [13.2 ; 17.4] | 0.163 |
| Vegetable protein | 4.5 [3.7 ; 5.1] | 4.3 [3.4 ; 5.3] | 0.738 |
| Animal protein | 9.8 [8.1 ; 11.3] | 11.0 [8.6 ; 13.1] | 0.285 |
| Carbohydrates | 45.1 [38 ; 50] | 44.6 [37.9 ; 51.8] | 0.738 |
| Disaccharides | 20.2 [15.3 ; 23.1] | 20.3 [15.3 ; 25.3] | 0.102 |
| Polysaccharides | 22.8 [17.5 ; 28.5] | 19.7 [16.2 ; 29.4] | 0.274 |
| Total fat | 34.1 [30.8 ; 39.2] | 34.2 [28.1 ; 39] | 0.442 |
| SFA | 13.2 [11.7 ; 15.2] | 12.5 [10.6 ; 15.7] | 0.087 |
| MUFA | 12.8 [11.4 ; 15.6] | 14 [11.1 ; 16.2] | 0.892 |
| PUFA | 4.8 [3.9 ; 5.8] | 4.5 [3.8 ; 5.8] | 0.097 |
| Alcohol | 4.3 [1.4 ; 8.5] | 3.4 [0.9 ; 7.9] | 0.781 |
| **Micronutrients** |  |  |  |
| Cholesterol (mg/d) | 308 [253 ; 376] | 274 [210 ; 368] | 0.109 |
| Calcium (mg/d) | 978 [725 ; 1400] | 843 [547 ; 1200] | 0.097 |
| Iron (mg/d) | 10.7 [8.6 ; 13.5] | 10.1 [8.1 ; 12.9] | 0.248 |
| Vitamin D | 1.8 [1.5 ; 2.7] | 2.3 [1.4 ; 3.4] | 0.285 |
| **Foods (g/day)** |  |  |  |
| Dairy | 195 [106 ; 280] | 148 [93 ; 226] | 0.163 |
| Red meat | 38 [26 ; 63] | 43 [27 ; 66] | 0.484 |
| Processed meat | 16 [8 ; 23] | 12 [5 ; 17] | 0.049 |
| Wholegrain | 34 [4 ; 63] | 12 [3 ; 54] | 0.353 |
| Fresh fruits | 127 [60 ; 251] | 136 [55 ; 226] | 0.713 |
| Fresh fruits + fresh juice | 176 [65 ; 269] | 156 [69 ; 284] | 0.944 |
| Any fruit and fruit juice | 222 [103 ; 400] | 226 [138 ; 340] | 0.426 |
| Vegetables | 130 [73 ; 191] | 125 [94 ; 157] | 0.325 |
| Fish, excluding fried | 20 [13 ; 32] | 27 [15 ; 46] | 0.272 |
| Fish, all | 26 [18 ; 40] | 37 [21 ; 55] | 0.172 |
| Ultraprocessed foods | 80 [43 ; 188] | 90 [29 ; 198] | 0.705 |
| **Compliance to guidelines (%)** ‖ |  |  |  |
| Fruits ≥2/day | 14 (34.2) | 14 (34.2) | 1.000 |
| Vegetables ≥3/day | 1 (2.4) | 0 (0) | 1.000 |
| Meat ≤5/week | 23 (56.1) | 23 (56.1) | 1.000 |
| Fish ≥1/week ^a^ | 28 (68.3) | 31 (75.6) | 0.581 |
| Fish ≥1/week ^b^ | 11 (26.8) | 18 (43.9) | 0.119 |
| Dairy ≥3/day | 1 (2.4) | 3 (7.3) | 0.625 |

^a^, excluding fried fish; ^b^, all fish; ‡ on 40 participants; † on 34 participants; **‖**, on 41 participants.

SFA, saturated fatty acids; MUFA, monounsaturated fatty acids; PUFA, polyunsaturated fatty acids; TEI, total energy intake.

Results are expressed as number of participants (column percentage) for categorical variables and as average±standard deviation or as median [interquartile range] for continuous variables. Between-group comparisons were performed using McNemar’s test for categorical variables and student’s t-test (*) or Wilcoxon’s sign test for continuous variables.

**Supplementary table 7.** Anthropometric, physical activity and dietary data before and after quitting, CoLaus study, Lausanne, Switzerland, obese participants.

|  | **Before** | **After** | **P-value** |
| --- | --- | --- | --- |
| Sample size | **14** | **14** |  |
| **Anthropometry** |  |  |  |
| Weight (kg) | 95.5 ± 18.9 | 98.3 ± 19 | 0.071 * |
| Body mass index (kg/m^2^) | 33.8 ± 4.4 | 35.2 ± 4.5 | 0.020 * |
| **Physical activity †** |  |  |  |
| Energy expenditure | 2713 [2533 ; 3795] | 2666 [2252 ; 3200] | 0.110 |
| Sedentarity (%) | 8 (61.5) | 9 (69.2) | 1.000 |
| Total energy intake (kcal) | 1838 [1116 ; 2401] | 1614 [1149 ; 2251] | 0.463 |
| **Macronutrients (g/d)** |  |  |  |
| Total protein | 69 [46 ; 88] | 58 [50 ; 85] | 0.426 |
| Vegetable protein | 17 [12 ; 26] | 15 [10 ; 25] | 0.670 |
| Animal protein | 49 [37 ; 53] | 46 [33 ; 57] | 0.502 |
| Carbohydrates | 153 [133 ; 307] | 175 [103 ; 255] | 0.502 |
| Disaccharides | 86 [57 ; 152] | 80 [63 ; 117] | 0.761 |
| Polysaccharides | 71 [61 ; 139] | 69 [43 ; 136] | 0.715 |
| Total fat | 71 [52 ; 95] | 55 [47 ; 82] | 0.463 |
| SFA | 31 [14 ; 38] | 20 [15 ; 29] | 0.153 |
| MUFA | 27 [20 ; 38] | 22 [17 ; 37] | 0.670 |
| PUFA | 9 [6 ; 13] | 9 [5 ; 11] | 0.326 |
| Alcohol | 10 [1 ; 17] | 8 [1 ; 27] | 0.658 |
| Fibre | 12 [9 ; 17] | 11 [7 ; 23] | 0.715 |
| **Macronutrients (% TEI)** |  |  |  |
| Total protein | 14.9 [13.3 ; 16.5] | 15.0 [13.5 ; 18.3] | 0.903 |
| Vegetable protein | 4.2 [3.0 ; 4.9] | 4.0 [3.6 ; 4.4] | 0.426 |
| Animal protein | 10.8 [7.9 ; 13.9] | 10.6 [9.1 ; 14.5] | 0.502 |
| Carbohydrates | 40.9 [34.7 ; 54.0] | 41.3 [38.5 ; 44.1] | 0.855 |
| Disaccharides | 22.4 [18.1 ; 28.9] | 23.3 [17.7 ; 30.1] | 0.761 |
| Polysaccharides | 19.0 [15.2 ; 26.8] | 18.3 [13.8 ; 22.0] | 0.326 |
| Total fat | 33.8 [29.6 ; 42.0] | 35.7 [31.5 ; 43.7] | 1.000 |
| SFA | 12.7 [9.8 ; 17.8] | 12.3 [10.7 ; 14.3] | 0.241 |
| MUFA | 14.6 [11.3 ; 18.3] | 14.6 [12.4 ; 16.9] | 0.670 |
| PUFA | 4.9 [4.3 ; 5.8] | 4.7 [4.0 ; 5.4] | 0.903 |
| Alcohol | 3.5 [0.8 ; 7.0] | 3.6 [0.6 ; 10.8] | 0.761 |
| **Micronutrients** |  |  |  |
| Cholesterol (mg/d) | 269 [146 ; 409] | 247 [198 ; 340] | 0.855 |
| Calcium (mg/d) | 1121 [539 ; 1688] | 898 [627 ; 1417] | 0.542 |
| Iron (mg/d) | 10.6 [6.9 ; 12.3] | 9.6 [6.3 ; 12.8] | 1.000 |
| Vitamin D | 1.9 [1.4 ; 3.7] | 1.9 [1.0 ; 3.0] | 0.583 |
| **Foods (g/day)** |  |  |  |
| Dairy | 206 [57 ; 478] | 211 [71 ; 305] | 0.414 |
| Red meat | 36 [19 ; 71] | 47 [30 ; 61] | 1.000 |
| Processed meat | 15 [3 ; 28] | 18 [10 ; 22] | 0.636 |
| Wholegrain | 19 [0 ; 51] | 13 [5 ; 44] | 0.581 |
| Fresh fruits | 190 [83 ; 320] | 250 [31 ; 370] | 0.326 |
| Fresh fruits + fresh juice | 190 [125 ; 320] | 341 [210 ; 370] | 0.040 |
| Any fruit and fruit juice | 300 [171 ; 380] | 352 [228 ; 553] | 0.110 |
| Vegetables | 114 [84 ; 178] | 106 [75 ; 181] | 0.952 |
| Fish, excluding fried | 19 [9 ; 26] | 19 [15 ; 32] | 0.542 |
| Fish, all | 23 [14 ; 39] | 32 [19 ; 46] | 0.853 |
| Ultraprocessed foods | 56 [7 ; 157] | 26 [11 ; 75] | 0.564 |
| **Compliance to guidelines (%)** |  |  |  |
| Fruits ≥2/day | 4 (30.8) | 8 (61.5) | 0.219 |
| Vegetables ≥3/day | 0 (0) | 0 (0) | NC |
| Meat ≤5/week | 6 (46.2) | 7 (53.9) | 1.000 |
| Fish ≥1/week ^a^ | 7 (53.9) | 9 (69.2) | 0.625 |
| Fish ≥1/week ^b^ | 4 (30.8) | 4 (30.8) | 1.000 |
| Dairy ≥3/day | 1 (7.7) | 0 (0) | 1.000 |

^a^, excluding fried fish; ^b^, all fish; †, on 13 participants.

SFA, saturated fatty acids; MUFA, monounsaturated fatty acids; PUFA, polyunsaturated fatty acids; TEI, total energy intake.

Results are expressed as number of participants (column percentage) for categorical variables and as average±standard deviation or as median [interquartile range] for continuous variables. Between-group comparisons were performed using McNemar’s test for categorical variables and student’s t-test (*) or Wilcoxon’s sign test for continuous variables.

**Supplementary table 8.** Changes between baseline and follow-up in anthropometry, physical activity and dietary intake between quitters and maintainers, and between quitters and never smokers, matched for gender and age, CoLaus study, Lausanne, Switzerland, women.

|  | **Quitters** | **Maintainers** | **Never smokers** | **P-value §** | **P-value ǂ** |
| --- | --- | --- | --- | --- | --- |
| Sample size | **56** | **56** | **56** |  |  |
| **Anthropometry** ‡ |  |  |  |  |  |
| Weight (kg) | 1.4 ± 3.7 | 0.8 ± 4.7 | -1.0 ± 5.0 | 0.517 * | 0.008 * |
| Body mass index (kg/m^2^) | 0.8 ± 1.4 | 0.5 ± 1.7 | -0.1 ± 2.0 | 0.429 * | 0.011 * |
| Energy expenditure (kcal) | -31 [-148 ; 132] | -36 [-207 ; 175] | -49 [-243 ; 137] | 0.808 | 0.370 |
| Total energy intake (kcal) | -31 [-351 ; 308] | 30 [-234 ; 373] | -56 [-513 ; 312] | 0.630 | 0.489 |
| **Macronutrients (g/d)** |  |  |  |  |  |
| Total protein | 3 [-10 ; 17] | 6 [-8 ; 16] | -4 [-19 ; 12] | 0.630 | 0.034 |
| Vegetable protein | 0 [-5 ; 4] | 1 [-3 ; 5] | -2 [-7 ; 4] | 0.454 | 0.198 |
| Animal protein | 4 [-6 ; 14] | 5 [-7 ; 14] | -5 [-9 ; 7] | 0.596 | 0.046 |
| Carbohydrates | -16 [-63 ; 38] | 3 [-34 ; 40] | -1 [-76 ; 52] | 0.607 | 0.780 |
| Disaccharides | -8 [-32 ; 26] | 0 [-30 ; 15] | 1 [-29 ; 24] | 0.997 | 0.509 |
| Polysaccharides | -6 [-30 ; 20] | 4 [-17 ; 33] | -2 [-56 ; 18] | 0.275 | 0.089 |
| Total fat | -1 [-16 ; 11] | 4 [-10 ; 20] | -2 [-15 ; 15] | 0.473 | 0.713 |
| SFA | -1 [-7 ; 5] | 2 [-3 ; 7] | -1 [-6 ; 7] | 0.244 | 0.619 |
| MUFA | 1 [-7 ; 7] | 2 [-4 ; 11] | -1 [-7 ; 6] | 0.439 | 0.499 |
| PUFA | 0 [-2 ; 2] | 0 [-1 ; 3] | -1 [-2 ; 1] | 0.546 | 0.286 |
| Alcohol | 0 [-1 ; 2] | 0 [-2 ; 3] | 0 [-1 ; 1] | 0.587 | 0.603 |
| Fibre | 0 [-3 ; 3] | 0 [-3 ; 4] | -1 [-6 ; 4] | 0.894 | 0.624 |
| **Macronutrients (% TEI)** |  |  |  |  |  |
| Total protein | 0.7 [-0.5 ; 2.9] | 0.6 [-1.4 ; 2.8] | -0.7 [-2.0 ; 1.3] | 0.568 | 0.007 |
| Vegetable protein | 0 [-0.6 ; 0.7] | 0 [-0.8 ; 0.9] | -0.2 [-1.0 ; 0.9] | 0.805 | 0.297 |
| Animal protein | 0.5 [-0.8 ; 3.2] | 0.9 [-1.8 ; 3.2] | -0.3 [-2.2 ; 2.2] | 0.805 | 0.041 |
| Carbohydrates | -2.9 [-7.3 ; 5.4] | -1.6 [-8.9 ; 0.8] | -2.6 [-7.8 ; 5.7] | 0.264 | 0.843 |
| Disaccharides | -1.0 [-6.3 ; 3.8] | -3.1 [-7.0 ; 0.4] | 1.3 [-4.2 ; 6.3] | 0.088 | 0.158 |
| Polysaccharides | -0.7 [-4.6 ; 4.4] | 1.2 [-4.9 ; 6.3] | -1.5 [-8.5 ; 4.8] | 0.671 | 0.131 |
| Total fat | 1.1 [-6.3 ; 6.7] | 1.8 [-3.0 ; 7.2] | 2.3 [-3.9 ; 6.8] | 0.086 | 0.392 |
| SFA | 0 [-2.7 ; 2.1] | 0.6 [-1.2 ; 3.5] | 0.7 [-2.0 ; 3.1] | 0.029 | 0.104 |
| MUFA | 0.4 [-2.9 ; 3.5] | 1.3 [-1.5 ; 4.2] | 0.8 [-1.8 ; 3.2] | 0.271 | 0.837 |
| PUFA | 0.1 [-0.7 ; 0.8] | 0.1 [-0.7 ; 1.3] | -0.2 [-1.2 ; 0.8] | 0.406 | 0.613 |
| Alcohol | 0 [-0.5 ; 1.1] | 0 [-1.0 ; 0.6] | 0 [-0.3 ; 0.4] | 0.796 | 0.587 |
| **Micronutrients** |  |  |  |  |  |
| Cholesterol (mg/d) | 27 [-46 ; 97] | 54 [-16 ; 116] | -11 [-71 ; 57] | 0.473 | 0.058 |
| Calcium (mg/d) | -49 [-285 ; 201] | 50 [-205 ; 288] | -1 [-236 ; 332] | 0.590 | 0.659 |
| Iron (mg/d) | 0.4 [-1.9 ; 2.2] | 0.6 [-1.4 ; 2.0] | -0.8 [-3.2 ; 1.6] | 0.818 | 0.036 |
| Vitamin D | 0.2 [-0.4 ; 1.2] | 0.1 [-0.6 ; 0.7] | -0.1 [-0.5 ; 0.8] | 0.055 | 0.357 |
| **Foods (g/day)** |  |  |  |  |  |
| Dairy | -40 [-81 ; 25] | 13 [-89 ; 66] | -4 [-83 ; 61] | 0.301 | 0.380 |
| Red meat | 0 [-9 ; 14] | 7 [-7 ; 23] | -8 [-23 ; 6] | 0.382 | 0.073 |
| Processed meat | 0 [-3 ; 2] | 0 [-4 ; 5] | 0 [-4 ; 4] | 0.693 | 0.504 |
| Wholegrain | 0 [-21 ; 22] | 0 [-16 ; 20] | 0 [-41 ; 16] | 0.786 | 0.590 |
| Fresh fruits | -3 [-122 ; 106] | -8 [-144 ; 42] | -4 [-119 ; 59] | 0.383 | 0.856 |
| Fresh fruits + fresh juice | 3 [-155 ; 143] | -7 [-144 ; 46] | 0 [-108 ; 147] | 0.264 | 0.724 |
| Any fruit and fruit juice | 1 [-193 ; 124] | -36 [-144 ; 38] | 13 [-143 ; 170] | 0.624 | 0.383 |
| Vegetables | 32 [-31 ; 85] | 7 [-43 ; 95] | -3 [-71 ; 69] | 0.958 | 0.142 |
| Fish, excluding fried | 2 [-3 ; 16] | 2 [-10 ; 14] | 0 [-10 ; 7] | 0.388 | 0.086 |
| Fish, all | 2 [-8 ; 16] | 2 [-10 ; 13] | 0 [-16 ; 12] | 0.383 | 0.061 |
| Ultraprocessed foods | -4 [-25 ; 5] | 1 [-35 ; 30] | 0 [-46 ; 15] | 0.894 | 0.521 |

‡ on 53 participants; §, comparing quitters to maintainers; **ǂ** comparing quitters to never smokers.

SFA, saturated fatty acids; MUFA, monounsaturated fatty acids; PUFA, polyunsaturated fatty acids; TEI, total energy intake.

For each participant, the difference between data collected in 2014-2017 and data collected in 2009-2012 were computed. Results are expressed as average±standard deviation or as median [interquartile range]. Between-group comparisons were performed using student’s t-test (*) or Wilcoxon sign test for continuous variables.

**Supplementary table 9.** Changes between baseline and follow-up in anthropometry, physical activity and dietary intake between quitters and maintainers, and between quitters and never smokers, matched for gender and age, CoLaus study, Lausanne, Switzerland, men.

|  | **Quitters** | **Maintainers** | **Never smokers** | **P-value §** | **P-value ǂ** |
| --- | --- | --- | --- | --- | --- |
| Sample size | **72** | **72** | **72** |  |  |
| **Anthropometry** ‡ |  |  |  |  |  |
| Weight (kg) | 2.7 ± 4.5 | -0.5 ± 4.8 | 0.2 ± 4.9 | <0.001 * | 0.003 * |
| Body mass index (kg/m^2^) | 1.0 ± 1.4 | 0 ± 1.6 | 0.2 ± 1.5 | <0.001 * | 0.002 * |
| Energy expenditure (kcal) | -67 [-402 ; 370] | 12 [-279 ; 308] | -121 [-429 ; 131] | 0.324 | 0.351 |
| Total energy intake (kcal) | -114 [-497 ; 335] | 150 [-308 ; 450] | -35 [-423 ; 310] | 0.080 | 0.598 |
| **Macronutrients (g/d)** |  |  |  |  |  |
| Total protein | -4 [-15 ; 13] | 2 [-15 ; 17] | -4 [-17 ; 13] | 0.480 | 0.358 |
| Vegetable protein | -2 [-7 ; 5] | 0 [-3 ; 5] | 0 [-7 ; 4] | 0.176 | 0.757 |
| Animal protein | -2 [-15 ; 10] | 2 [-15 ; 17] | 1 [-10 ; 12] | 0.723 | 0.189 |
| Carbohydrates | -10 [-62 ; 42] | 10 [-35 ; 51] | -10 [-61 ; 40] | 0.272 | 0.834 |
| Disaccharides | 0 [-26 ; 27] | 6 [-24 ; 27] | -2 [-27 ; 22] | 0.327 | 0.993 |
| Polysaccharides | -9 [-40 ; 25] | 5 [-23 ; 26] | 0 [-35 ; 27] | 0.148 | 0.641 |
| Total fat | -6 [-20 ; 16] | 2 [-11 ; 17] | -1 [-16 ; 12] | 0.067 | 0.182 |
| SFA | -3 [-10 ; 3] | -1 [-7 ; 7] | -1 [-9 ; 5] | 0.027 | 0.130 |
| MUFA | -2 [-7 ; 8] | 1 [-4 ; 7] | -2 [-5 ; 6] | 0.182 | 0.426 |
| PUFA | -1 [-3 ; 1] | 0 [-2 ; 4] | 0 [-3 ; 3] | 0.052 | 0.100 |
| Alcohol | 1 [-7 ; 4] | 0 [-4 ; 7] | 0 [-2 ; 3] | 0.537 | 0.530 |
| Fibre | 0 [-4 ; 4] | 1 [-2 ; 5] | -1 [-4 ; 2] | 0.180 | 0.962 |
| **Macronutrients (% TEI)** |  |  |  |  |  |
| Total protein | 0.1 [-1.8 ; 2.5] | -0.2 [-2.3 ; 1.7] | 0.5 [-1.4 ; 2.0] | 0.182 | 0.778 |
| Vegetable protein | 0 [-0.9 ; 0.6] | -0.1 [-0.6 ; 0.9] | -0.2 [-0.8 ; 0.7] | 0.508 | 0.694 |
| Animal protein | 0.6 [-1.6 ; 3.5] | -0.6 [-2.7 ; 1.6] | 0.3 [-1.3 ; 2.3] | 0.154 | 0.774 |
| Carbohydrates | -0.1 [-4.5 ; 3.6] | 0.1 [-4.8 ; 5.6] | -1.0 [-5.9 ; 3.7] | 0.727 | 0.401 |
| Disaccharides | 0.7 [-2.1 ; 4.5] | -0.6 [-4.4 ; 4.0] | 0.4 [-3.7 ; 4.3] | 0.171 | 0.308 |
| Polysaccharides | -0.8 [-6.2 ; 3.2] | -0.4 [-5.8 ; 5.2] | -1.5 [-5.9 ; 4.2] | 0.583 | 0.953 |
| Total fat | 0.3 [-3.4 ; 3.5] | -1.6 [-4.7 ; 3.3] | 0.9 [-3.5 ; 5.3] | 0.641 | 0.473 |
| SFA | -0.8 [-2.9 ; 1.4] | -0.9 [-2.5 ; 1.4] | 0.4 [-2.4 ; 1.7] | 0.702 | 0.287 |
| MUFA | 0.3 [-1.1 ; 2.2] | -0.4 [-2.2 ; 2.0] | 0.4 [-1.9 ; 2.9] | 0.473 | 0.657 |
| PUFA | 0 [-0.7 ; 0.8] | 0.1 [-0.7 ; 1.0] | 0.2 [-0.6 ; 1.5] | 0.459 | 0.217 |
| Alcohol | -0.3 [-2.1 ; 1.0] | -0.1 [-2 ; 3.7] | 0.1 [-0.6 ; 1.2] | 0.379 | 0.169 |
| **Micronutrients** |  |  |  |  |  |
| Cholesterol (mg/d) | -34 [-85 ; 70] | 20 [-33 ; 103] | 10 [-66 ; 72] | 0.015 | 0.111 |
| Calcium (mg/d) | -109 [-462 ; 139] | 90 [-321 ; 377] | -72 [-364 ; 214] | 0.041 | 0.426 |
| Iron (mg/d) | -0.3 [-2.3 ; 2] | 0.2 [-2 ; 2.6] | -0.4 [-2.7 ; 2.3] | 0.690 | 0.967 |
| Vitamin D | -0.1 [-0.6 ; 1] | 0.6 [-0.4 ; 1.6] | 0.1 [-0.7 ; 1.7] | 0.099 | 0.456 |
| **Foods (g/day)** |  |  |  |  |  |
| Dairy | -17 [-81 ; 38] | -4 [-58 ; 66] | -6 [-64 ; 53] | 0.392 | 0.372 |
| Red meat | 0 [-17 ; 23] | -6 [-38 ; 17] | 0 [-21 ; 24] | 0.276 | 0.837 |
| Processed meat | -1 [-12 ; 6] | -1 [-11 ; 8] | 2 [-6 ; 7] | 0.526 | 0.256 |
| Wholegrain | 0 [-15 ; 22] | 2 [-7 ; 28] | 0 [-21 ; 6] | 0.284 | 0.859 |
| Fresh fruits | 15 [-31 ; 70] | 18 [-18 ; 126] | -13 [-71 ; 49] | 0.144 | 0.526 |
| Fresh fruits + fresh juice | 32 [-28 ; 80] | 22 [-26 ; 133] | -19 [-83 ; 49] | 0.675 | 0.129 |
| Any fruit and fruit juice | 37 [-25 ; 126] | 6 [-71 ; 123] | -25 [-100 ; 79] | 0.386 | 0.013 |
| Vegetables | -12 [-38 ; 34] | -4 [-39 ; 42] | 10 [-33 ; 57] | 0.883 | 0.321 |
| Fish, excluding fried | 2 [-7 ; 14] | 7 [-4 ; 23] | 0 [-13 ; 13] | 0.276 | 0.140 |
| Fish, all | 2 [-12 ; 17] | 8 [-6 ; 22] | -2 [-15 ; 16] | 0.425 | 0.168 |
| Ultraprocessed foods | -5 [-56 ; 39] | -2 [-50 ; 24] | -4 [-43 ; 33] | 0.825 | 0.847 |

§, comparing quitters to maintainers; **ǂ** comparing quitters to never smokers.

SFA, saturated fatty acids; MUFA, monounsaturated fatty acids; PUFA, polyunsaturated fatty acids; TEI, total energy intake.

For each participant, the difference between data collected in 2014-2017 and data collected in 2009-2012 were computed Results are expressed as average±standard deviation or as median [interquartile range]. ‡ N=69 participants. Between-group comparisons were performed using student’s t-test (*) or Wilcoxon sign test for continuous variables.

**Supplementary table 10.** Changes between baseline and follow-up in anthropometry, physical activity and dietary intake according to time since quitting, CoLaus study, Lausanne, Switzerland.

|  | **≤1 year** | **>1 and ≤2 years** | **>2 years** | **P-value** |
| --- | --- | --- | --- | --- |
| Sample size | **18** | **15** | **35** |  |
| **Anthropometry** ‡ |  |  |  |  |
| Weight (kg) | 2.8 ± 4.8 | 3.1 ± 3.4 | 2.3 ± 5.0 | 0.832 * |
| Body mass index (kg/m^2^) | 1.3 ± 1.8 | 1.2 ± 1.3 | 1.0 ± 1.5 | 0.753 * |
| Energy expenditure **†** | -133 [-324 ; 118] | -107 [-462 ; 370] | 105 [-86 ; 390] | 0.146 |
| Total energy intake (kcal) | -211 [-373 ; 27] | 10 [-299 ; 468] | 108 [-528 ; 411] | 0.488 |
| **Macronutrients (g/d)** |  |  |  |  |
| Total protein | 1 [-21 ; 21] | 12 [-4 ; 41] | 2 [-18 ; 18] | 0.177 |
| Vegetable protein | 0 [-4 ; 3] | 1 [-5 ; 5] | 0 [-3 ; 4] | 0.941 |
| Animal protein | 1 [-15 ; 15] | 5 [-2 ; 30] | 0 [-17 ; 13] | 0.182 |
| Carbohydrates | -18 [-57 ; 42] | -8 [-50 ; 57] | 8 [-59 ; 67] | 0.683 |
| Disaccharides | -7 [-24 ; 14] | -5 [-25 ; 24] | 7 [-27 ; 44] | 0.461 |
| Polysaccharides | 3 [-32 ; 32] | -6 [-29 ; 37] | -6 [-27 ; 20] | 0.885 |
| Total fat | -12 [-20 ; -1] | 7 [-7 ; 22] | -6 [-31 ; 23] | 0.202 |
| SFA | -5 [-9 ; 1] | 1 [-4 ; 12] | -4 [-12 ; 5] | 0.163 |
| MUFA | -3 [-7 ; 0] | 3 [-4 ; 10] | -3 [-12 ; 9] | 0.310 |
| PUFA | -2 [-2 ; -1] | 1 [-1 ; 2] | -1 [-4 ; 2] | 0.064 |
| Alcohol | 0 [-3 ; 3] | -2 [-8 ; 1] | 0 [-3 ; 5] | 0.483 |
| Fibre | 0 [-4 ; 2] | 3 [-3 ; 7] | 0 [-2 ; 3] | 0.622 |
| **Macronutrients (% TEI)** |  |  |  |  |
| Total protein | 0.6 [-2.1 ; 4.1] | 1.6 [-0.3 ; 4.8] | 0.5 [-1.5 ; 2.5] | 0.183 |
| Vegetable protein | 0.2 [-0.9 ; 1] | 0 [-1.5 ; 1.3] | 0.2 [-0.5 ; 0.9] | 0.780 |
| Animal protein | 0.7 [-3.2 ; 3.9] | 3.4 [-0.2 ; 5.1] | 0 [-2.0 ; 2.8] | 0.126 |
| Carbohydrates | -0.7 [-5.7 ; 3.4] | -4.4 [-10.3 ; 3.5] | 4.4 [-2.6 ; 7.9] | 0.082 |
| Disaccharides | -0.6 [-4.8 ; 3.7] | -2.2 [-5.9 ; -0.4] | 1.9 [-1.4 ; 8.9] | 0.043 |
| Polysaccharides | 0.4 [-6.9 ; 4.9] | -0.5 [-8.3 ; 7.5] | -0.4 [-3.4 ; 4.9] | 0.908 |
| Total fat | -2.4 [-4.9 ; 4.4] | 3.3 [-3.7 ; 8.6] | -2.6 [-7.6 ; 3.6] | 0.111 |
| SFA | -1.2 [-2.1 ; 0.9] | 2.3 [-1.4 ; 3.9] | -1.3 [-3.7 ; 1.5] | 0.109 |
| MUFA | -0.1 [-1.3 ; 2.2] | 0.6 [-1.8 ; 3.2] | -0.5 [-3.1 ; 1.8] | 0.327 |
| PUFA | -0.3 [-1.1 ; 0.5] | 0 [-0.4 ; 0.8] | 0 [-1.7 ; 0.6] | 0.236 |
| Alcohol | 0.1 [-1.3 ; 2.2] | -1.1 [-3.9 ; 0.3] | 0 [-2.1 ; 1.1] | 0.196 |
| **Micronutrients** |  |  |  |  |
| Cholesterol (mg/d) | -2 [-64 ; 31] | 94 [-60 ; 171] | 0 [-85 ; 83] | 0.179 |
| Calcium (mg/d) | 24 [-462 ; 140] | 66 [-159 ; 113] | -99 [-485 ; 249] | 0.486 |
| Iron (mg/d) | -0.3 [-2.0 ; 2.3] | 1 [-1.5 ; 4.2] | 0.4 [-2.0 ; 2.4] | 0.575 |
| Vitamin D | 0.2 [-0.3 ; 0.5] | 0 [-1.6 ; 0.7] | 0.2 [-0.5 ; 1.4] | 0.310 |
| **Foods (g/day)** |  |  |  |  |
| Dairy | -32 [-74 ; 2] | -1 [-58 ; 20] | -17 [-80 ; 48] | 0.413 |
| Red meat | -5 [-15 ; 30] | 5 [-8 ; 30] | 1 [-24 ; 18] | 0.644 |
| Processed meat | 0 [-4 ; 3] | -1 [-12 ; 1] | 0 [-13 ; 7] | 0.615 |
| Wholegrain | 0 [-17 ; 18] | 0 [-11 ; 19] | 2 [-17 ; 25] | 0.766 |
| Fresh fruits | -10 [-102 ; 29] | 37 [-188 ; 251] | 46 [-4 ; 114] | 0.085 |
| Fresh fruits + fresh juice | -14 [-35 ; 40] | 63 [34 ; 251] | 45 [-10 ; 216] | 0.082 |
| Any fruit and fruit juice | -25 [-102 ; 8] | 34 [-65 ; 158] | 81 [-45 ; 216] | 0.033 |
| Vegetables | 28 [-20 ; 112] | 10 [-32 ; 38] | -17 [-61 ; 42] | 0.229 |
| Fish, excluding fried | -2 [-3 ; 5] | 1 [-7 ; 15] | 3 [-8 ; 16] | 0.590 |
| Fish, all | 2 [-8 ; 13] | 5 [-5 ; 15] | 2 [-12 ; 23] | 0.842 |
| Ultraprocessed foods | -2 [-14 ; 45] | 0 [-50 ; 30] | -4 [-36 ; 59] | 0.786 |

SFA, saturated fatty acids; MUFA, monounsaturated fatty acids; PUFA, polyunsaturated fatty acids; TEI, total energy intake.

‡ for 64 participants (16 / 15 / 33); **†** for 48 participants (14 / 10 / 24).

For each participant, the difference between data collected in 2014-2017 and data collected in 2009-2012 were computed. Results are expressed as average±standard deviation or as median [interquartile range]. Between-group comparisons were performed using analysis of variance (*) or Kruskal-Wallis test for continuous variables.

**Supplementary table 11.** Correlations between changes between baseline and follow-up in anthropometry and dietary and time since quitting or weight changes, CoLaus study, Lausanne, Switzerland.

|  | **Time since quitting** | **P-value** | **Weight changes** | **P-value** |
| --- | --- | --- | --- | --- |
| Sample size | **68** |  | **122** |  |
| Weight (kg) ‡ | -0.033 | 0.802 | - |  |
| Body mass index (kg/m^2^) ‡ | -0.026 | 0.844 | - |  |
| Total energy intake (kcal) | 0.040 | 0.748 | -0.033 | 0.797 |
| **Macronutrients (g/d)** |  |  |  |  |
| Total protein | -0.072 | 0.579 | 0.042 | 0.747 |
| Vegetable protein | -0.047 | 0.719 | -0.081 | 0.531 |
| Animal protein | -0.072 | 0.580 | 0.034 | 0.795 |
| Carbohydrates | -0.005 | 0.969 | -0.043 | 0.739 |
| Disaccharides | 0.036 | 0.781 | -0.072 | 0.577 |
| Polysaccharides | -0.075 | 0.561 | -0.045 | 0.727 |
| Total fat | -0.005 | 0.972 | -0.083 | 0.522 |
| SFA | -0.027 | 0.834 | -0.125 | 0.333 |
| MUFA | -0.022 | 0.867 | -0.069 | 0.593 |
| PUFA | 0.062 | 0.633 | 0.019 | 0.883 |
| Alcohol | 0.100 | 0.438 | -0.118 | 0.360 |
| Fibre | -0.049 | 0.704 | -0.127 | 0.327 |
| **Macronutrients (% TEI)** |  |  |  |  |
| Total protein | -0.158 | 0.220 | 0.014 | 0.917 |
| Vegetable protein | 0.006 | 0.965 | 0.027 | 0.835 |
| Animal protein | -0.178 | 0.167 | -0.038 | 0.771 |
| Carbohydrates | 0.131 | 0.310 | 0.108 | 0.404 |
| Disaccharides | 0.120 | 0.351 | -0.040 | 0.760 |
| Polysaccharides | -0.008 | 0.950 | 0.091 | 0.482 |
| Total fat | -0.060 | 0.642 | -0.164 | 0.202 |
| SFA | -0.068 | 0.601 | -0.142 | 0.272 |
| MUFA | -0.068 | 0.599 | -0.122 | 0.344 |
| PUFA | 0.017 | 0.898 | -0.009 | 0.947 |
| Alcohol | 0.003 | 0.980 | -0.010 | 0.940 |
| **Micronutrients** |  |  |  |  |
| Cholesterol (mg/d) | -0.082 | 0.525 | 0.002 | 0.987 |
| Calcium (mg/d) | -0.012 | 0.924 | -0.137 | 0.290 |
| Iron (mg/d) | -0.036 | 0.784 | 0.104 | 0.419 |
| Vitamin D | 0.067 | 0.604 | -0.089 | 0.493 |
| **Foods (g/day)** |  |  |  |  |
| Dairy | 0.109 | 0.399 | -0.086 | 0.508 |
| Red meat | -0.060 | 0.646 | 0.182 | 0.156 |
| Processed meat | -0.051 | 0.695 | -0.200 | 0.120 |
| Wholegrain | 0.093 | 0.475 | -0.070 | 0.590 |
| Fresh fruits | 0.135 | 0.296 | -0.181 | 0.159 |
| Fresh fruits + fresh juice | 0.066 | 0.611 | -0.068 | 0.602 |
| Any fruit and fruit juice | 0.171 | 0.185 | 0.041 | 0.752 |
| Vegetables | -0.207 | 0.107 | -0.173 | 0.179 |
| Fish, excluding fried | 0.013 | 0.918 | 0.033 | 0.801 |
| Fish, all | -0.062 | 0.631 | 0.009 | 0.942 |
| Ultraprocessed foods | -0.077 | 0.554 | 0.090 | 0.489 |

‡ for 64 participants. Results are expressed as Spearman correlation coefficient.
