# Supplementary figures and images for: No changes in dietary intake after quitting smoking; a prospective study in Switzerland
Source: BMC Nutr. 2021 Jul 14;7:34. doi: 10.1186/s40795-021-00440-4 (PMC8278689; doi:10.1186/s40795-021-00440-4)

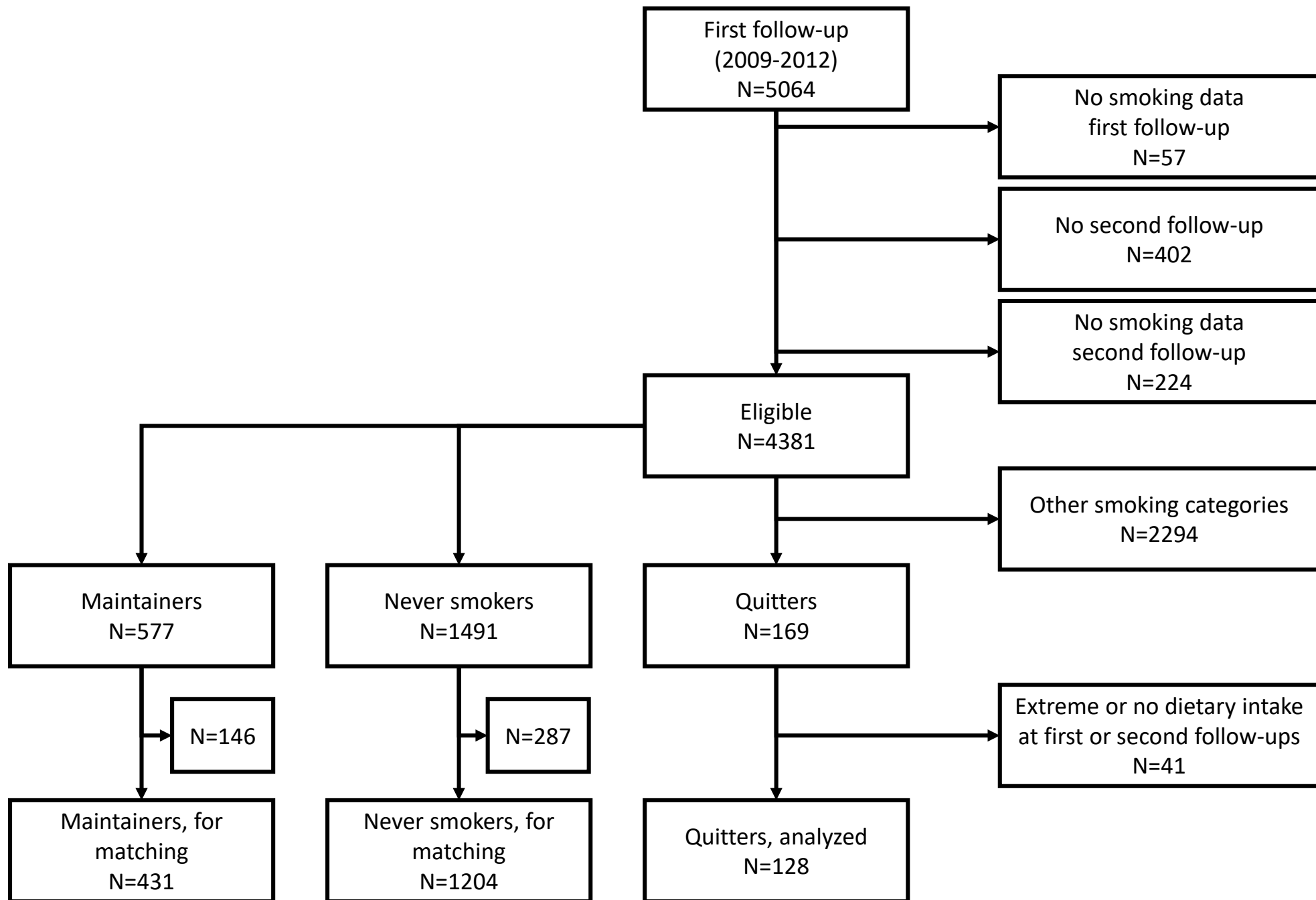

Supplement: Supplementary file 2 — Additional file 2. Supplementary Figure 1. [file 40795_2021_440_MOESM2_ESM.pdf]
